# Binary‐Solvent‐Programmed Single‐Step Inkjet Printing of Self‐Confined Micro‐Inlaid OLED Arrays

**DOI:** 10.1002/smtd.70769

**Published:** 2026-06-15

**Authors:** JaeWoo Park, Wonsun Kim, Kimin Lee, Sui Yang, Eun Ha Choi, Byoungchoo Park

**Affiliations:** ^1^ Materials Science and Engineering Fulton Schools of Engineering Arizona State University Tempe Arizona USA; ^2^ Department of Plasma‐Bio Display Kwangwoon University Seoul South Korea; ^3^ Department of Electrical and Biological Physics Kwangwoon University Seoul South Korea

**Keywords:** µ‐OLED arrays, Hansen solubility parameter, mixed‐solvent interaction, phase separation, single‐step inkjet printing, micro‐inlaid OLED

## Abstract

Inkjet printing offers a mask‐free route to large‐area electronics, yet achieving uniformity in micron‐scale organic light‐emitting diode (µ‐OLED) arrays remains challenging. Presented here is a photolithography‐free, solvent‐programmed, single‐step inkjet micro‐inlay process in which lateral phase separation self‐confines each emissive pixel. Guided by solubility parameters, a trichloromethane (TCM)/1,2‐dichloroethane (DCE) binary solvent is designed to optimize interactions among the solvents, emissive solutes, and the poly(4‐vinylpyridine) (P4VP) underlayer. Micro‐Raman mapping, cross‐sectional SEM, and AFM phase analysis support lateral phase separation between the emissive region and the displaced P4VP phase, selective restructuring of P4VP while preserving the underlying transport layer, and no detectable nanoscale phase segregation within the emissive interior, yielding self‐confined pixels of approximately 100 µm. High‐speed imaging shows that the blend yields reproducible 180 dpi arrays without jetting instability or nonuniform deposition. Green µ‐OLED arrays printed with the blend achieve a peak luminance of 2400 cd m^−2^, a peak current efficiency of 3.5 cd A^−1^, and a peak external quantum efficiency of 1.0%. The figure of merit and luminance uniformity improve by 2.6‐ and 6.9‐fold, and by 3.9‐ and 2.9‐fold, respectively, relative to neat TCM and neat DCE. This strategy enables scalable fabrication of flexible and three‐dimensional conformal OLED platforms.

## Graphical abstract

Solvent‐programmed micro‐inlay inkjet printing enables lithography‐free, high‐resolution μ‐OLED arrays in a single step. A thermodynamically tuned 3:1 (w/w) chloroform/dichloroethane blend not only drives interfacial phase separation between the solute and polymers but also stabilizes droplet ejection, forming rim‐confined emissive domains in one pass. The approach delivers reproducible 180‐dpi patterning (∼100 µm pixels) with bright, efficient emission, supporting scalable display fabrication.

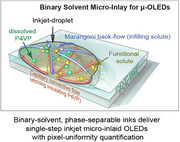

## 1 Introduction

Inkjet printing has emerged as a highly promising, mask‐free technique for the fabrication of large‐area, high‐resolution optoelectronics [1, 2, 3, 4, 5, 6, 7, 8, 9, 10, 11, 12, 13, 14]. It allows for the selective deposition of functional materials, eliminating the need for complex photolithographic patterning and reducing material waste [1, 2, 3, 4, 5, 6, 7, 8, 9, 10, 11, 12, 13, 14]. This additive manufacturing approach is particularly attractive for organic light‐emitting diodes (OLEDs), which require precise micron‐scale patterning over large substrate areas [10, 11, 12, 15, 16, 17, 18, 19, 20, 21, 22, 23, 24, 25, 26, 27, 28, 29]. However, despite its potential, critical technical challenges have hindered the widespread adoption of inkjet printing in OLED manufacturing [21, 22, 23, 24, 25, 26, 29]. Conventional inkjet methodologies are challenged when tasked with simultaneously achieving the requisite lateral precision, device efficiency, and pixel‐to‐pixel uniformity [21, 22, 23, 24, 25, 26, 29], which are imperative for next‐generation micro‐displays with sub‐100‐µm pixel dimensions. The primary obstacle is to achieve lateral confinement without sacrificing droplet placement accuracy and/or uniformity. Without physical boundaries, ink droplets tend to spread uncontrollably when deposited onto substrates. This spreading can lead to the well‐known “coffee‐ring” effect, where evaporation‐driven capillary flows carry dissolved functional materials toward the perimeter of the droplet, resulting in a non‐uniform deposition and unpredictable pixel geometries [29, 30, 31, 32, 33, 34]. Consequently, the resulting pixels exhibit poor uniformity and reproducibility, compromising device performance and array‐level yields.

Traditionally, this lateral containment issue has been addressed by using lithographically defined polymer “banks”, such as polyimide‐ or acrylate‐based structures, to confine the printed droplets physically [34, 35]. However, these methods increase the processing complexity and cost due to multiple lithographic steps and can induce potential contamination risks from residual materials. An alternative approach is “inkjet etching”, which uses sequential printing passes [29, 30, 31, 32, 33, 34]. First, a solvent droplet dissolves an insulating polymer to create a via hole or shallow well. Then, in a second pass, emissive inks fill the via hole. While this two‐step process eliminates the need for photolithographic banks, it introduces variability in the etch depth and perimeter geometry and, critically, instills stringent alignment requirements [29, 30, 31, 32, 33, 34]. These factors significantly undermine the pixel‐level uniformity and reproducibility.

Recently, single‐step self‐alignment strategies utilizing spontaneous lateral phase separation within a single deposited droplet have attracted attention [36, 37, 38]. Previous studies have shown that droplets containing chloroform (trichloromethane, TCM) and small‐molecule emissive blends can locally dissolve a poly(4‐vinylpyridine) (P4VP) insulating layer, forming a bank‐like hole, and refill it through internal phase separation during a single printing event [36, 37, 38]. This process produces self‐confined emissive islands without relying on pre‐patterned banks and achieves pixel sizes of around 100 µm with improved simplicity and efficiency. However, several significant limitations remain unaddressed. The low boiling point (∼61°C) of TCM causes rapid evaporation at the nozzle orifice, resulting in frequent clogging of the nozzle and unstable droplet ejection. Additionally, uncontrolled swelling of the polymer matrix causes substantial variability between devices, limiting practical scalability [36, 39]. These shortcomings underscore the central role of the carrier solvent in determining the performance capabilities of inkjet‐printed OLEDs. Solvent volatility, viscosity, surface tension, and solubility govern the jetting stability, polymer interaction, and phase‐separation kinetics. Therefore, we shifted our research focus from optimizing single‐solvent formulations to designing binary‐solvent mixtures, hypothesizing that blending solvents with complementary physicochemical properties would mitigate jetting instability issues while enabling controlled solvent‐polymer‐solute interactions.

In this study, we systematically evaluate a binary‐solvent strategy that combines TCM with 1,2‐dichloroethane (DCE) given its high boiling point at an optimized 3:1 (w/w) ratio. At room temperature (25°C), TCM (boiling point ≈ 61°C) exhibits a substantially higher vapor pressure (≈ 197 mmHg) and slightly lower viscosity (≈ 0.58 cP) than DCE (boiling point ≈ 83°C; vapor pressure ≈ 78.9 mmHg; viscosity ≈ 0.79 cP) [40, 41, 42, 43], suggesting that their combination enables the tuning of the evaporation kinetics and rheological properties for uniform inkjet printing. A Flory–Huggins interaction parameter (χ) analysis based on a Hansen solubility parameters (HSPs, δs) analysis, corroborated by micro‐Raman mapping, demonstrates that this binary system optimally balances the affinity and selective interactions between the emissive small‐molecule solute and the insulating P4VP matrix [14, 36, 37, 39, 44, 45]. The balanced mixed solvent enables stable printing, yielding uniform emissive micro‐inlays with a surface roughness of less than 2 nm via the effective lateral phase separation of P4VP.

The use of the mixed solvent produced micron‐scale OLED (µ‐OLED) arrays with superior performance and uniformity compared to single‐solvent inks. Specifically, the arrays exhibited luminance values that were approximately 1.4‐ and 2.7‐fold higher than those from neat‐TCM‐ and neat‐DCE‐based arrays, respectively, along with substantial improvements in the external quantum efficiency (*EQE*). Moreover, the luminance uniformity of the mixed‐solvent based µ‐OLED pixels increased by approximately 3.9‐ and 2.9‐fold relative to devices made with neat TCM and neat DCE, respectively. These results highlight the importance of binary solvent engineering for achieving precise, reproducible, and high‐performance µ‐OLED arrays. By eliminating the need for masks, photolithography, and dual‐pass alignment, the mixed‐solvent‐based single‐step inkjet micro‐inlay technique offers a scalable, versatile, and robust manufacturing route for next‐generation OLED micro‐displays and other solution‐processed optoelectronics [26, 28, 46, 47].

## 2 Results and Discussion

### 2.1 Thermodynamic Tuning of Mixed‐Solvent Interactions for Controlled Phase Separation and Micro‐Inlay Formation

**FIGURE 1:**
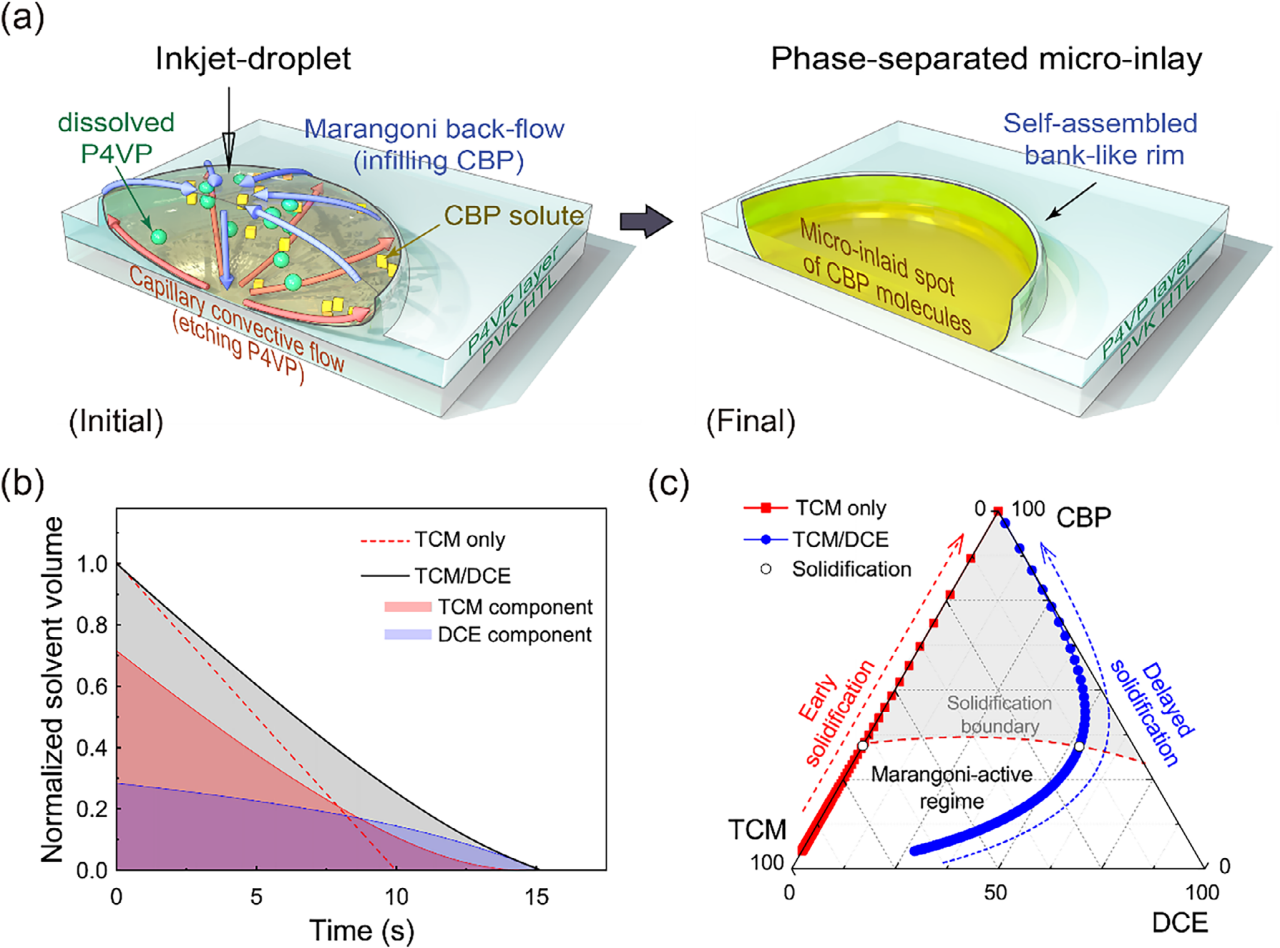
Solvent‐programmed single‐step formation of a micro‐inlaid emissive structure: (a) Schematic illustration of drying‐induced micro‐inlay formation, in which capillary‐driven outward flow and Marangoni back‐flow generate a centrally confined CBP region surrounded by a self‐assembled bank‐like rim. (b) Simulated evaporation kinetics of neat TCM and a 3:1 (w/w) TCM/DCE binary solvent, showing a prolonged liquid‐state lifetime of the mixed solvent due to preferential TCM evaporation. (c) Conceptual ternary compositional pathway of the TCM–DCE–CBP system, indicating delayed crossing of the solidification boundary and the resulting extension of the liquid‐state processing window.

First, we revisit the formation mechanism of the single‐step inkjet‐printed micro‐inlay illustrated in Figure 1 in order to explain how the mixed‐solvent approach creates self‐confined emissive spots. The inks in this study contain a wide‐band‐gap emitter (4,4′‐bis(carbazol‐9‐yl)biphenyl, CBP) dissolved in a binary solvent mixture of high‐volatility TCM and relatively less volatile DCE at a weight ratio of 3:1. The ink droplets (approximately 0.1 nL) are deposited via an inkjet onto a substrate pre‐coated with a phase‐separable insulating P4VP layer on top of a glass‐supported indium‐tin oxide (ITO) electrode. Additional functional layers, for example, poly(3,4‐ethylenedioxythiophene):poly(styrene sulfonate) (PEDOT:PSS, hole‐injection layer (HIL)) and/or poly(N‐vinylcarbazole) (PVK, hole‐transport layer (HTL)), can be incorporated beneath the P4VP layer without altering the micro‐inlay mechanism described herein.

When the inkjet‐ejected droplet drops onto the substrate, the mixed solvent in the droplet selectively swells and locally dissolves the underlying insulating P4VP layer. The mixed solvent of the TCM and DCE then initiates an outward, capillary‐driven flow, i.e., the coffee‐ring effect. This transports the dissolved polymer toward the perimeter of the droplet and creates a transient liquid trench [7, 8, 29, 30, 31, 32, 33, 34, 48]. Concurrently, differential evaporation rates between the TCM and DCE generate a Marangoni back‐flow directed toward the droplet center [13, 14, 31, 48]. This flow efficiently concentrates the dissolved CBP solute at the center position. Thus, these two counteracting flows simultaneously displace the P4VP layer from the central region and deposit the emissive CBP within the newly formed cavity (Figure 1a, left).

As the solvent mixture evaporates, the polymer retracts and solidifies into a robust, self‐ assembled rim, while the CBP precipitates into the recessed, polymer‐free interior. The resulting self‐confined emissive spot exhibits a uniformly smooth surface enclosed by the phase‐separated P4VP rim (Figure 1a, right). This single‐step, lithography‐free mechanism integrates polymer removal, solute transport, and localized deposition within a single droplet‐printing event, thereby enabling µ‐OLED array fabrication without pre‐patterned banks or subsequent alignment steps.

To elucidate the physicochemical origin of the improved film uniformity enabled by the binary solvent system, the droplet‐drying behavior was analyzed using a simplified coupled kinetic–thermodynamic model that combines the evaporation kinetics with compositional–pathway evolution. In this framework, the time‐dependent evaporation behavior of a sessile droplet was evaluated using a diffusion‐limited model based on Raoult's law under a pinned contact‐line condition, and the corresponding compositional evolution was interpreted using a conceptual ternary phase diagram of the TCM–DCE–CBP system [49, 50, 51, 52]. Here, the solidification boundary is treated as a semi‐quantitative indicator of the onset of solute vitrification based on χ‐derived miscibility trends, rather than as a rigorously defined thermodynamic binodal boundary. This model was introduced to capture, in a simplified but physically transparent manner, how preferential solvent evaporation changes the approach to solidification during ink drying.

As shown in Figure 1b, pure TCM evaporates rapidly and reaches the solidification regime within a short time window. In contrast, the mixed solvent (TCM:DCE = 3:1 w/w) undergoes preferential evaporation of the more volatile TCM component, progressively enriching the remaining liquid in DCE and producing a transient DCE‐rich fluid regime with a prolonged evaporation tail. Using a threshold‐extension criterion, the mixed solvent was estimated to maintain the liquid state for an additional 2.85–4.33 s relative to neat TCM; at the representative 10% threshold, the liquid‐window times were 9.12 s for neat TCM and 12.83 s for the TCM/DCE mixture.

Based on this kinetic evolution, the conceptual ternary diagram in Figure 1c describes how the drying pathway changes in the composition space. Because in situ measurements of the transient ternary composition during droplet drying are experimentally challenging, the present representation is intended to provide a physically consistent visualization linking the evaporation kinetics with the phase evolution. In the pure‐TCM case, the trajectory proceeds nearly directly toward the solute‐rich region and intersects the solidification boundary at an early stage of drying. Such premature solidification restricts internal fluid motion and increases the likelihood of localized solute accumulation and structural heterogeneity. In contrast, the binary solvent follows a curved compositional trajectory toward a DCE‐rich region before reaching the solidification boundary, thereby remaining longer in the single‐phase liquid region. This delayed solidification widens the liquid‐state processing window, allowing the Marangoni‐driven back‐flow to counterbalance capillary‐driven outward transport during the drying process [50, 51]. The direction of the Marangoni flow is consistent with the preferential depletion of TCM during drying, as TCM has a lower surface tension (γ ≈ 27.1 mN m^−1^) than DCE (γ ≈ 32.8 mN m^−1^).

Consequently, solute redistribution can proceed within an extended liquid‐state regime, suppressing premature aggregation and promoting the formation of a more uniform amorphous micro‐inlaid emissive layer (EML). These results indicate that binary‐solvent engineering acts primarily as a kinetic regulator of the liquid‐to‐solid transition, beyond equilibrium miscibility considerations, thus providing a mechanistic explanation for the enhanced pixel uniformity observed in the µ‐OLED arrays.

The key to forming well‐defined, micro‐inlaid CBP spots depends heavily on the thermodynamics of lateral phase separation between the emissive CBP solute and the insulating P4VP matrix. The Flory–Huggins interaction parameter χ conveniently quantifies the driving force and represents the enthalpic cost of mixing two components [14, 45]. For blends of two components *i* and *j*, the χ value can be approximated by the equation χ_*i* − *j*_ = (*V_i_V_j_*)^0.5^ /(*RT*)·(δ_*i*_ − δ_*j*_)^2^, where *R* is the gas constant, *T* is the absolute temperature, and *V_i_* and δ_*i*_ are the monomeric molar volume and solubility parameter of component *i*, respectively [53, 54, 55]. Interaction parameters exceeding 0.5 (χ_*i* − *j*_ > 0.5) generally indicate thermodynamically unfavorable mixing and significant phase separation [14, 45].

Table 1 summarizes the dispersive (δ_d_), polar (δ_p_), and hydrogen‐bonding (δ_h_) contributions of the HSP values for all compounds used in this study. Owing to discrepancies in the literature regarding the reported HSP values of P4VP (19–25 MPa^1/2^), we re‐estimated the δ values for P4VP using the Microsoft Excel Solver optimization tool [56]. This yielded a total HSP (δ_tot_) of approximately 27.4 MPa^1/2^, consistent with the high affinity of P4VP for strongly polar solvents, such as glycerol carbonate. Using these HSP values, the estimated χ values (at 300 K) are approximately χ_CBP − P4VP_ = 1.9 for CBP–P4VP and higher (approximately 3.6) for other functional solutes, such as 2‐(4‐tert‐butylphenyl)‐5‐(4‐biphenylyl)‐1,3,4‐oxadiazole (PBD), 1,3,5‐tri(m‐pyridin‐3‐ylphenyl)benzene (TmPyPB), and tris(2‐phenylpyridine)iridium(III) (Ir(ppy)_3_), vs. P4VP. These high χ values confirm strong thermodynamic driving forces that promote phase segregation between P4VP and functional solutes, including CBP, upon droplet deposition.

**TABLE 1:** Hansen solubility parameters (δs) and molar volumes (*V*_s_s) of the functional materials investigated, determined using the solver add‐in of Microsoft Excel [56].

Compound	*δ*_d_ (MPa^1/2^)	*δ*_p_ (MPa^1/2^)	*δ*_h_ (MPa^1/2^)	*δ* (MPa^1/2^)	*V* (cm^3^ mol^−1^)
P4VP	17.2	14.3	15.9	27.4	107.7
CBP	17.5	7.7	6.5	20.2	404.8
PBD	17.6	8.3	7.9	21.0	321.2
TmPyPB	17.7	8.4	10.0	22.0	394.1
Ir(ppy)_3_	18.0	12.3	7.2	23.0	403.9
PVK	17.2	8.3	6.7	20.2	162.4
TCM	17.8	3.1	5.7	18.9	79.6
DCE	17.0	8.2	7.0	20.1	78.8
Mixed TCM/DCE (3:1 w/w)	17.6	4.6	6.1	19.1	81.1

*δ*_d_: Hansen dispersion solubility parameter; *δ*_p_: polar solubility parameter; *δ*_h_: hydrogen bonding solubility parameter. *δ* = (*δ*_d_^2^ + *δ*_p_^2^+ *δ*_h_^2^)^0.5^.

Next, to evaluate the compatibility between the mixed solvent and the phase‐separable polymer matrix of the functional solute quantitatively, we estimated the HSPs of the 3:1 (w/w) ratio TCM/DCE blend. Converting to mole fractions (*X*_TCM_ = 0.71 and *X*_DCE_ = 0.29) and undertaking the mole‐fraction weighting of the individual solvent components yielded composite HSPs; i.e., δ_i/j, d/p/h_ =  *X*_i_ · δ_i, d/p/h_ + *X*_j_ · δ_j, d/p/h_. The resulting values were δ_TCM/DCE,d_ = 17.6 MPa^1/2^, δ_TCM/DCE,p_ = 4.6 MPa^1/2^, and δ_TCM/DCE,h_ = 6.1 MPa^1/2^, thereby presenting a total solubility parameter, δ_TCM/DCE,tot_, of approximately 19.1 MPa^1/2^. Further refinement of the polymer‐solvent affinity was achieved with these values through the Flory‐Huggins interaction parameter χ, which was calculated using the extended formulation of $\chi_{\mathrm{solvent}-\mathrm{solute}}=\frac{V_{s}}{RT}\;\left[{\left(\delta_{\mathrm{solvent},d}-\delta_{\mathrm{solute},d}\right)}^{2}+0.25{\left(\delta_{\mathrm{solvent},p}-\delta_{\mathrm{solute},p}\right)}^{2}+0.25\right.$ $\left.{\left(\delta_{\mathrm{solvent},h}-\delta_{\mathrm{solute},h}\right)}^{2}\right]$ [57]. Here, *V*_s_ represents the molar volume of the solvent. The estimated density and molar volume for the TCM/DCE mixture are ρ_TCM/DCE_ = 1.408 g cm^−3^ and *V*_TCM/DCE_ ≈ 81 cm^3^ mol^−1^, respectively. With *T* set to 300 K, the analysis yields the following χ values: χ_TCM − P4VP_ = 1.8, χ_DCE − P4VP_ = 0.9, and χ_TCM/DCE − P4VP_ = 1.6 for the TCM/DCE blend with the 3:1 (w/w) ratio. This intermediate value for the binary blend allows for controlled swelling and partial dissolution of the polymer, which is sufficient to promote localized etching of the P4VP insulating layer without over‐dissolution or uncontrolled spreading. In contrast, the emissive small‐molecule CBP shows strong solubility in all three solvents: χ_solvent − CBP_ = 0.2 with TCM, nearly zero with DCE, and 0.1 with the TCM/DCE blend. This reflects excellent solute solvation throughout droplet drying.

Thus, tuning the engineered mixture of solvents to the appropriate interaction parameters, i.e., χs, governs the emergence of a stable hierarchy of oppositely directed flows. The high χ mismatch between the mixed solvent and the P4VP polymer induces outward capillary transport of the polymer. Meanwhile, the low χ_TCM/DCE − CBP_ value ensures that the CBP remains dissolved and is driven inward to the droplet center by a Marangoni back‐flow as the TCM evaporates more rapidly than the DCE. Importantly, the present micro‐inlay mechanism should not be regarded as intrinsically restricted to solvents with boiling points below approximately 100°C. The governing parameter is not the absolute boiling point itself but rather the accessible drying timescale that permits delayed solidification together with internal fluid redistribution. In the TCM/DCE system studied here, this requirement is satisfied by combining a highly volatile component with a less volatile one, thereby extending the liquid‐phase lifetime relative to neat TCM while maintaining printable jetting behavior. In principle, solvent systems with substantially higher boiling points could also provide a suitable processing window for controlled lateral phase separation; however, such systems would require concurrent optimization of the viscosity, surface tension, wetting behavior, and jetting stability for the specific printhead configuration [58, 59]. Therefore, the broader implication of the present results is a kinetic design principle based on evaporation‐pathway engineering rather than a solvent‐specific rule defined solely by the boiling point. This dual‐function behavior, which promotes the formation of a polymer bank‐like hole while retaining solute confinement, is unique to the engineered TCM/DCE system. The interplay among volatility, solubility, and flow symmetry enables the lithography‐free patterning of sharply defined, uniformly smooth emissive islands, which is difficult to attain using either solvent alone.

### 2.2 Influence of the Solvent Composition on the Topography of Inkjet‐Printed Pixels on Functional Multilayers

**FIGURE 2:**
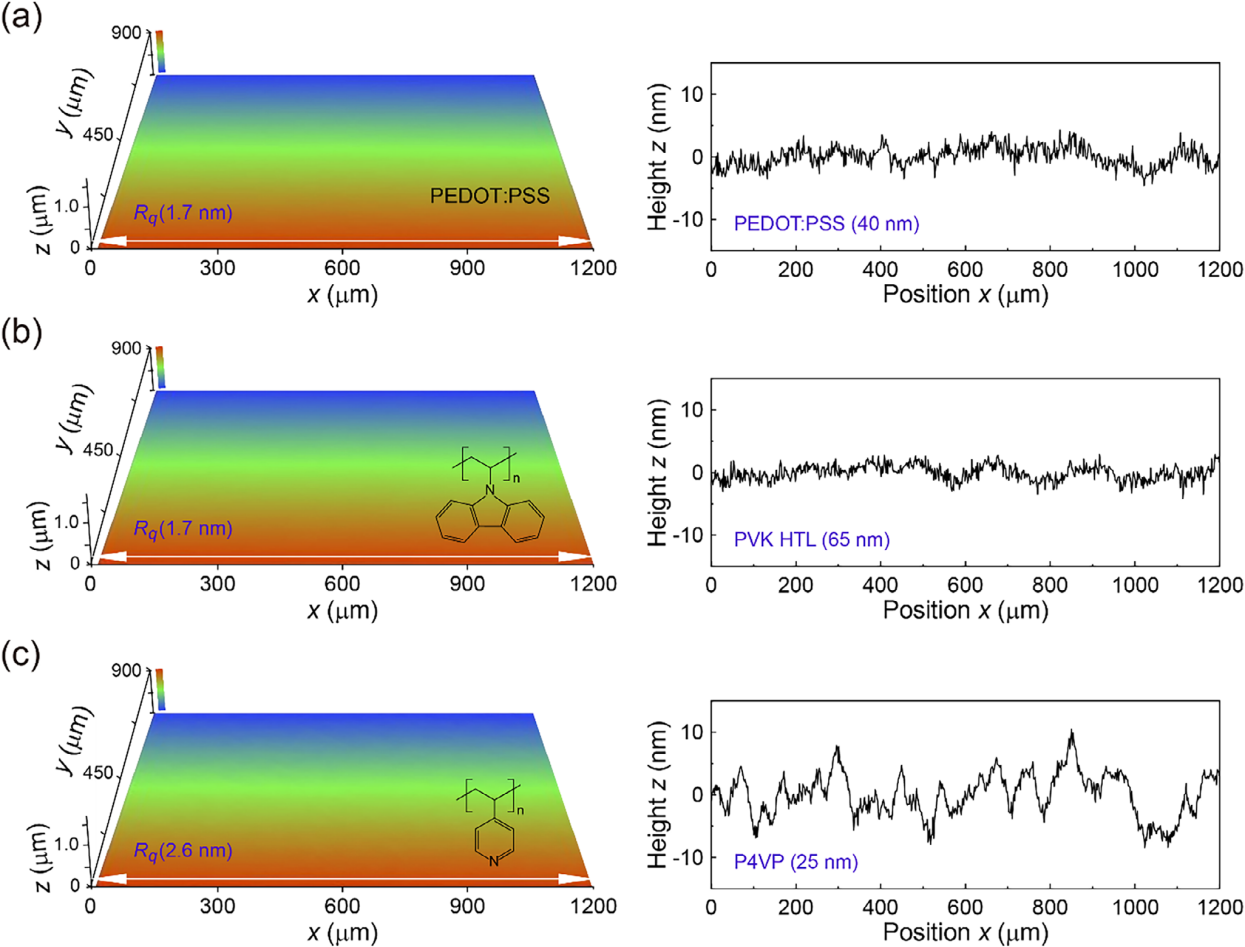
Surface topography of the multilayer stack characterized by optical 3D profilometry. Each panel presents a height map (left) and a corresponding line scan (right): (a) a 40‐nm‐thick PEDOT:PSS film spin‐coated onto ITO glass with an *R*_q_ value of 1.7 nm, (b) a 65‐nm‐thick PVK layer with an *R*_q_ value of 1.7 nm deposited atop the PEDOT:PSS/ITO stack, and (c) a 25‐nm‐thick P4VP overlayer with an *R*_q_ value of 2.6 nm. The molecular structures of PVK and P4VP are shown as insets in the height maps. The scan area for each measurement is 1200 × 900 µm^2^.

The interfacial morphology of each functional layer strongly influences the wetting behavior and solvent‐polymer interactions, thereby determining the fidelity of micro‐inlaid pixel formation in inkjet‐printed optoelectronic devices. To ensure uniform phase‐separation dynamics and the reliable confinement of emissive domains, the underlying substrate stack must be smooth and homogeneous. Figure 2 shows 3D optical profilometry images confirming that each layer in the P4VP/PVK HTL/PEDOT:PSS HIL/ITO architecture has a root‐mean‐square (RMS) surface roughness of less than 3 nm. For instance, the 40‐nm‐thick PEDOT:PSS HIL, spin‐coated on ITO/glass, has a featureless surface with RMS roughness of 1.7 nm (Figure 2a). This indicates minimal defect density and high planarity at the anode interface. Depositing the 65‐nm‐thick PVK HTL atop the PEDOT:PSS HIL preserves this morphological uniformity, maintaining an identical RMS roughness of 1.7 nm (Figure 2b). Adding a 25‐nm‐thick P4VP overlayer increases the surface roughness slightly to 2.6 nm (Figure 2c). However, it remains within the threshold necessary for consistent droplet spreading and confinement behavior. Across a lateral scan area of 1.2 × 0.9 mm^2^, no spike‐like defects or interfacial discontinuities are observed, indicative of the high spatial uniformity of the multilayer stack. Such low surface roughness indicates high film quality, which is essential for well‐defined solvent‐polymer interactions and a predictable microfluidic flow during printing [60]. Specifically, the smooth and chemically compatible P4VP surface enables the TCM/DCE solvent blend to dissolve the layer selectively, promoting symmetric inward solute transport and yielding smooth emissive islands confined within self‐assembled polymer rims. Therefore, maintaining a homogeneous surface morphology at each interface is essential for producing repeatable micro‐inlay geometries and achieving high‐fidelity patterning in these inkjet‐printed micro‐pixel arrays, as shown below.

**FIGURE 3:**
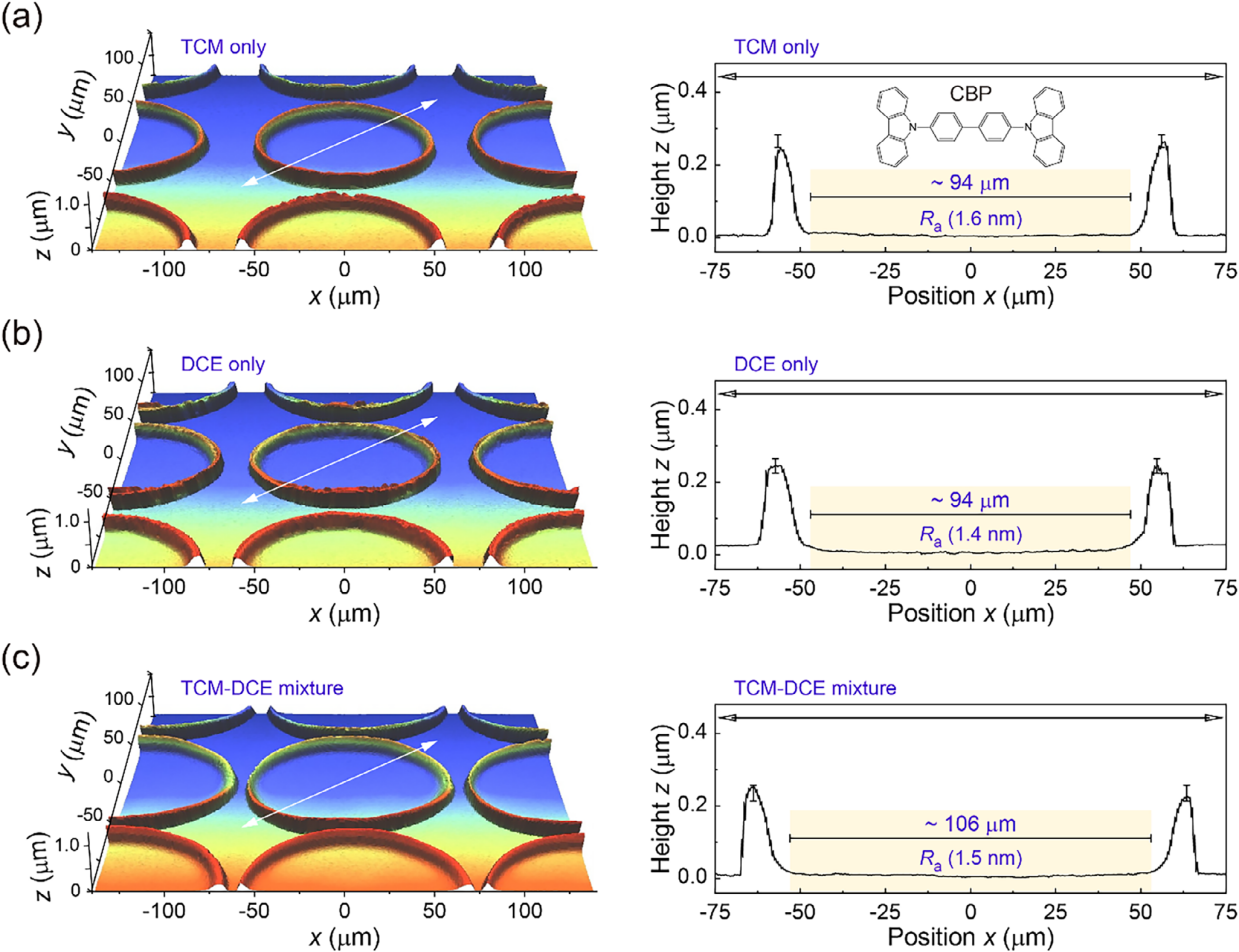
Solvent‐dependent morphology of single‐step inkjet‐printed CBP micro‐pixels. Left panels show 3D optical profilometry height maps, and right panels show representative line profiles across the emissive region of pixels printed at 180 dpi using CBP‐based phase‐separable inks formulated with (a) neat TCM, (b) neat DCE, and (c) a 3:1 (w/w) TCM/DCE binary solvent. All inks contained identical CBP concentrations and were deposited on multilayer substrates (P4VP/PVK/PEDOT:PSS on ITO glass). The profiles exhibit a central plateau corresponding to the CBP emissive island and annular rim peaks at the perimeter. Error bars indicate pixel‐to‐pixel variation in the rim height (*N* = 20). The central emissive region remained comparably smooth in all cases, with *R*_a_ values of 1.6 nm (TCM), 1.4 nm (DCE), and 1.5 nm (TCM/DCE). The inset shows the molecular structure of CBP.

Based on the confirmed nanometer‐scale smoothness of the P4VP/PVK/PEDOT:PSS stack (Figure 2), we subsequently investigated how the solvent composition affects the geometry of inkjet‐printed pixels. Micro‐spot arrays were printed at 180 dots per inch (dpi) using emissive inks dissolved in three different solvent systems: pure TCM, pure DCE, and a 3:1 (w/w) ratio TCM/DCE binary blend. All formulations were deposited onto identical P4VP/PVK/PEDOT:PSS multilayers to isolate the effects of each solvent selected for use. Figure 3 shows representative three‐dimensional profilometry maps (left) and corresponding cross‐sectional height profiles (right) for each ink system formulated with neat TCM, neat DCE, and a TCM/DCE (3:1, w/w) binary solvent (Figure 3a–c). Regardless of the solvent type, the droplets self‐organized into a distinctive morphological motif: a circularly confined feature surrounded by a well‐defined annular rim. This characteristic pattern formation suggests that solvent‐induced transient swelling of the top P4VP layer, combined with evaporation‐driven flow mechanisms, in this case the Marangoni effect, will enable the spatial redistribution of ink components as the droplets dry. Importantly, the cross‐sectional profiles in Figure 3 represent the composite surface topography of the displaced P4VP rim and the printed CBP‐based emissive island formed on the PVK underlayer, rather than the rim geometry alone. Therefore, the central plateau should be interpreted as an indirect indicator of the relative thickness distribution of the CBP‐based EML within the active pixel region. Under neat‐TCM conditions, the short liquid‐phase lifetime limits internal redistribution and is consistent with stronger capillary‐driven outward accumulation during drying, resulting in a less favorable interior profile (*R*_a_ ≈ 1.6 nm) (Figure 3a). In contrast, the TCM/DCE binary solvent preserves the fluid state for a longer period, thereby allowing further an additional internal redistribution before solidification, consistent with the flatter central plateau observed in Figure 3. In this sense, the mixed‐solvent condition is interpreted as providing a more favorable thickness profile within the emissive region (*R*_a_ ≈ 1.5 nm) rather than merely generating a more regular peripheral contour (Figure 3c). This interpretation is also consistent with the drying‐mediated transport framework discussed above, in which prolonged liquid‐state evolution can mitigate strong peripheral accumulation through solvent‐dependent internal flow, plausibly including Marangoni‐assisted back‐flow [50, 51].

Despite their shared overall morphological structure, quantitative differences in the peripheral rim geometry were observed under different solvent conditions. Neat TCM yielded taller rim peaks (266 ± 18 nm) and narrower rims (full width at half maximum, FWHM ≈ 5.5 µm), indicating rapid solvent evaporation and more localized interaction with the P4VP surface (Figure 3a). In contrast, neat DCE, with its higher boiling point and lower vapor pressure, produced broader rims (FWHM ≈ 7.0 µm) with slightly lower peak heights (245 ± 20 nm), reflecting enhanced lateral swelling caused by prolonged solvent–polymer contact (Figure 3b). The mixed TCM/DCE solvent system showed intermediate characteristics, with a rim height of 235 ± 22 nm and an FWHM of ≈ 6.2 µm (Figure 3c). The small error bars at the rim peaks in Figure 3 indicate the pixel‐to‐pixel variation in the rim height measured across multiple pixels (*N* = 20).

Although the peripheral rim geometry varies with solvent condition, the central emissive region remains comparably smooth across all three systems, with similar roughness values (*R*_a_ ≈ 1.4–1.6 nm). This indicates that the solvent‐dependent morphological differences are expressed primarily at the perimeter, whereas the local surface quality of the active region is largely preserved. Accordingly, the mixed‐solvent condition is interpreted as providing a more favorable internal deposition profile and thickness distribution within the emissive region, rather than merely yielding a more regular peripheral contour.

### 2.3 Multiscale Structural and Phase Analysis of Solvent‐Dependent Micro‐Inlay Formation

#### 2.3.1 Spatial Raman Mapping of Solvent‐Dependent Lateral Phase Separation

**FIGURE 4:**
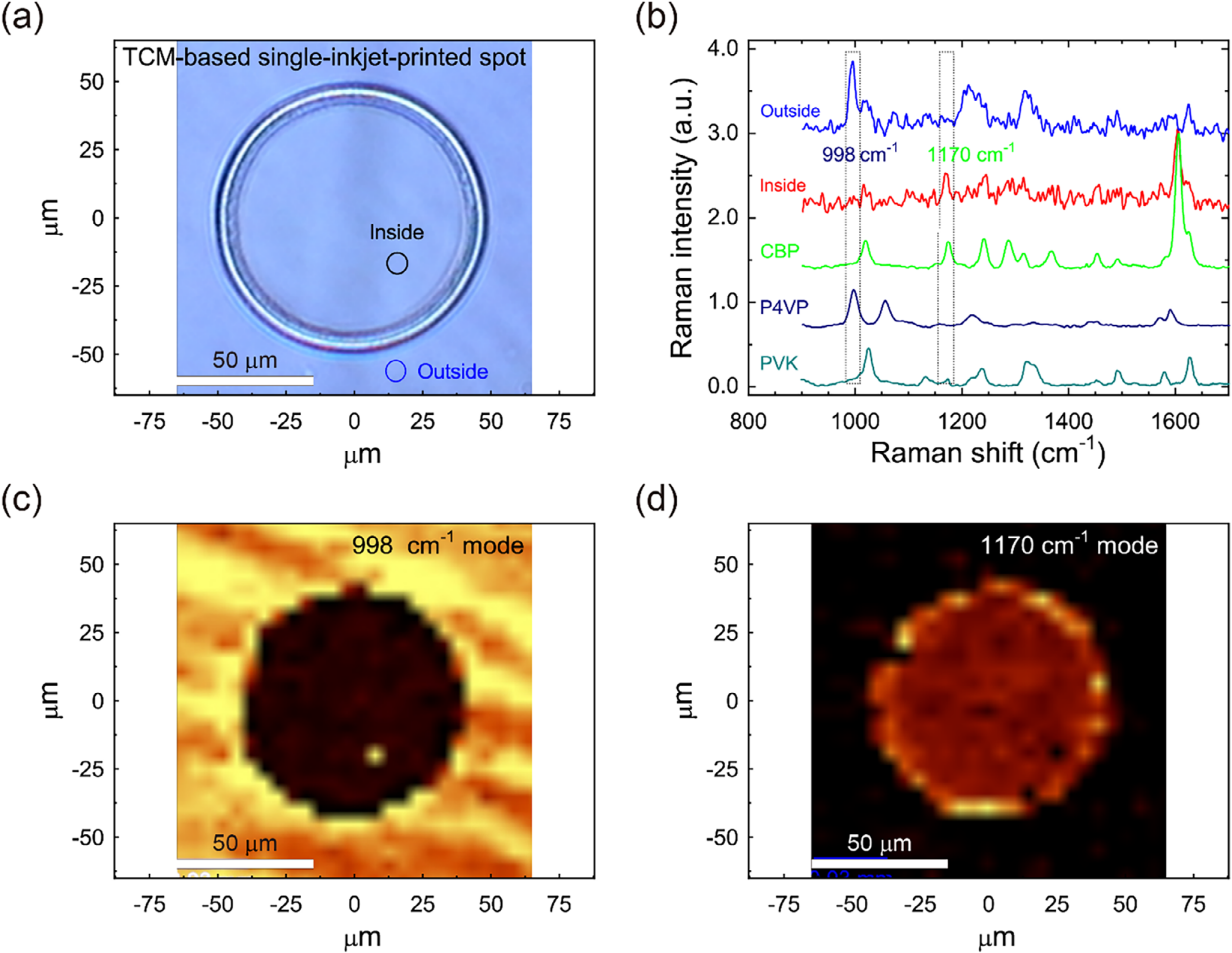
Results of Raman spectroscopic analyses of the material distributions within a single inkjet‐printed CBP spot: (a) Optical microscope image of a representative CBP spot printed using TCM‐based ink on a P4VP/PVK stack on a silver‐coated substrate. (b) Point micro‐Raman spectra collected from the inside and outside regions of the spot under 632.8 nm excitation (1 mW). Reference spectra of pristine CBP, P4VP, and PVK are included for spectral assignment and validation. (c, d) Raman intensity maps showing the spatial distributions of (c) P4VP (998 cm^−^^1^ mode) and (d) CBP (1170 cm^−^^1^ mode) within the same printed region. It should be noted that the isolated bright point near the bottom‐right corner of the dark circular region in the Raman map (Figure 4c) is an artifact caused by spectral noise spikes, not a genuine Raman signal.

To verify the lateral phase separation of the chemical compounds induced by the solvent‐programmed inkjet printing process, we used micro‐Raman spectroscopy to determine the spatial distribution of the individual molecular components within the printed pixels. Specifically, we examined whether the morphology of the single‐step printed pixel, consisting of a circular island surrounded by a well‐defined annular rim, reflects spatial separation between the emissive solute and the sacrificial polymer. Micro‐Raman spectroscopy was utilized to examine micro‐pixels printed at 180 dpi using a CBP ink dissolved in neat TCM on a multilayer substrate consisting of a 25‐nm‐thick P4VP layer, a 65‐nm‐thick PVK HTL, and a 100‐nm‐thick silver underlayer. The silver underlayer was introduced to enhance the Raman signal intensity through surface‐enhanced Raman scattering (SERS)‐like effects. Figure 4a shows an optical micrograph of a representative pixel selected for analysis. This pixel exhibits the characteristic circular spot morphology, with a diameter of approximately 100 µm, consistent with profilometric observations (Figure 3a). Micro‐Raman spectra were acquired from two representative locations: inside the central spot and from the surrounding annular region. Figure 4b shows that the Raman signal obtained from the outer region is dominated by a sharp peak at 998 cm^−1^, which corresponds to the ring‐breathing mode of P4VP. In contrast, the spectrum recorded from the center region of the spot reveals a distinct peak at 1170 cm^−1^ attributable to the in‐plane C─H bending vibration of CBP [36, 61, 62, 63]. Notably, these two modes show negligible spectral overlap between the inner and outer regions, indicating that the molecular species remain spatially segregated after the inkjet printing process.

To visualize the distribution of the chemical components further, spatially resolved Raman maps were constructed by integrating the intensity of the 998 and 1170 cm^−1^ modes across the entire pixel area (Figure 4c,d, respectively). The P4VP map (998 cm^−1^) shows intense Raman signals localized exclusively outside the rim, while the CBP map (1170 cm^−1^) reveals strong signals confined inside the spot region. These complementary spatial patterns confirm that the two components were clearly separated during the droplet drying process without residual intermixing above the detection threshold. These Raman mapping results provide direct spectroscopic evidence that capillary and Marangoni flows, in conjunction with solvent‐polymer‐solute interactions, drive the physical redistribution of chemical species [37]. The resulting phase‐separated structure, with a CBP‐rich island surrounded by a P4VP boundary, validates the thermodynamic and microflow‐based mechanisms of the single‐step, solvent‐induced micro‐inlay approach proposed here.

**FIGURE 5:**
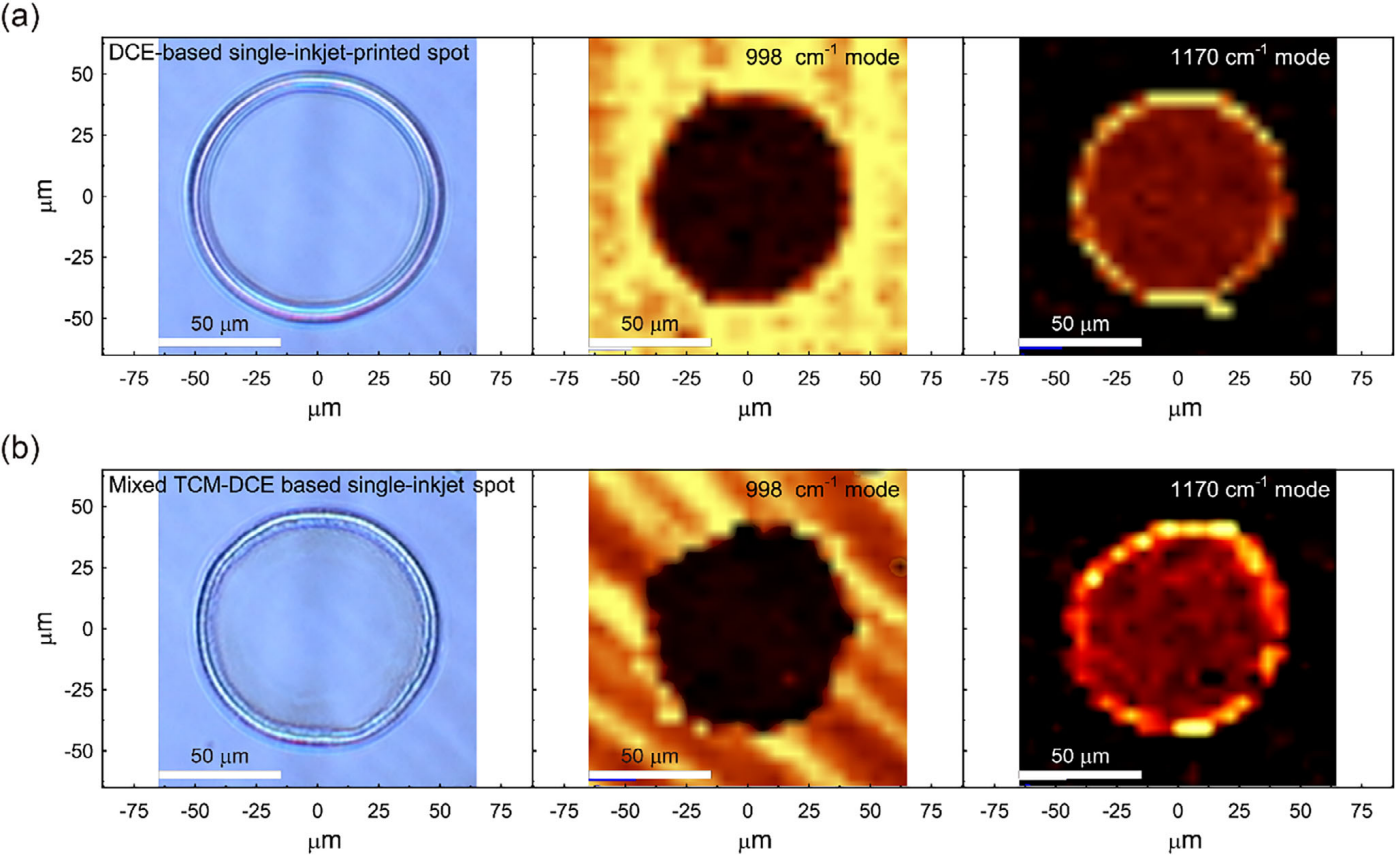
Solvent‐dependent lateral redistribution of CBP revealed by micro‐Raman mapping. Optical microscope images (left) and Raman intensity maps of the 998 cm^−1^ P4VP mode (middle) and the 1170 cm^−1^ CBP mode (right) for CBP spots printed using (a) DCE‐only and (b) TCM/DCE (3:1 w/w ratio) inks on P4VP/PVK/PEDOT:PSS/ITO substrates. The spatial contrast between the P4VP‐rich rim and CBP‐rich core highlights the solvent‐induced lateral phase separation. All Raman measurements were performed under 632.8 nm excitation at 1 mW of laser power.

Next, to evaluate the robustness and generality of the previously described solvent‐directed lateral phase‐separation mechanism, we expanded the Raman spectroscopic analysis to include not only neat TCM but also neat DCE and the 3:1 (w/w) ratio TCM/DCE blend. These analyses show that the formation of laterally segregated micro‐inlaid structures is not limited to specific solvent systems. Rather, these structures emerge from the engineered interaction landscape of the solvent‐polymer‐solute system. As shown in Figure 5a, spots printed with inkjet technology using neat‐DCE‐based ink exhibit a morphology similar to that of spots printed using neat‐TCM‐based ink. Despite its distinct physicochemical properties, including a higher boiling point (≈84°C), an increased hydrogen‐bonding capacity (δ_h_ = 7.0 MPa^1/2^), and a slightly higher total HSP (δ_tot_ ≈ 20.1 MPa^1/2^), DCE produces similar circular spots with sharply defined annuli and consistent diameters close to 100 µm. Spatial micro‐Raman spectroscopic mapping confirmed the phase‐separation behavior. The Raman intensity map of the 998 cm^−1^ P4VP ring‐breathing mode shows a strong signal outside the spot boundary, indicating that the polymer is displaced laterally outside the rim. In contrast, the region inside the spot boundary lacks a P4VP signal. These results confirm that the solubility mismatch with the P4VP polymer layer (Δδ_tot_ ≈ 7 MPa^1/2^) relative to DCE remains within the range required to induce transient polymer swelling and localized dissolution. Conversely, the 1170 cm^−1^ C─H in‐plane bending mode, which is characteristic of CBP, appears exclusively within the spot interior and is entirely absent from the annular region. These spatially anti‐correlated distributions confirm that effective lateral phase separation occurs between the P4VP and CBP phases, even with the more slowly evaporating, more polar DCE solvent. This ability of DCE to produce similar inlay architectures implies that the coupling of volatility and polymer solvency critically controls the jetting fidelity and phase separation. This insight supports the binary‐solvent strategy that leverages the complementary properties of TCM and DCE to optimize inlay formation.

Next, Figure 5b shows that the 3:1 TCM/DCE blend preserves the self‐inlay architecture while sharpening the edge of the CBP‐rich emissive island within the P4VP boundary. The optical micrograph reveals a printed spot with a diameter of ≈ 100 µm bounded by a well‐defined annulus. Raman intensity maps at 998 cm^−1^ (P4VP) and 1170 cm^−1^ (CBP) clearly show mutually exclusive spatial distributions, consistent with observations of neat solvents [64, 65]. Specifically, the P4VP signal is confined to the outer region of the annular rim, while the CBP signal remains strictly localized within the central spot. No cross‐contamination or bleed‐over is detected, underscoring the high chemical purity of the phase‐separated regions. This behavior is attributed to the optimized solubility profile, viscosity, and evaporation kinetics of the mixed solvent, which collectively promote clean lateral segregation during the printing process.

The TCM/DCE blend, with a total HSP (δ_tot_ ≈ 19.1 MPa^1/2^) intermediate to the corresponding neat solvents, exhibits a solubility mismatch with the P4VP layer (δ_P4VP_ ≈ 27.4 MPa^1/2^) sufficient to induce controlled, localized swelling without excessive dissolution. As the droplet dries, the more volatile TCM component drives a rapid Marangoni back‐flow toward the droplet center, while the DCE component with a higher boiling point maintains the viscosity and suppresses any instability of the ink. Together, these effects yield reproducible lateral phase separation and high‐resolution pixel fidelity.

#### 2.3.2 Cross‐Sectional SEM Analysis of Solvent‐Dependent Vertical Structural Selectivity

**FIGURE 6:**
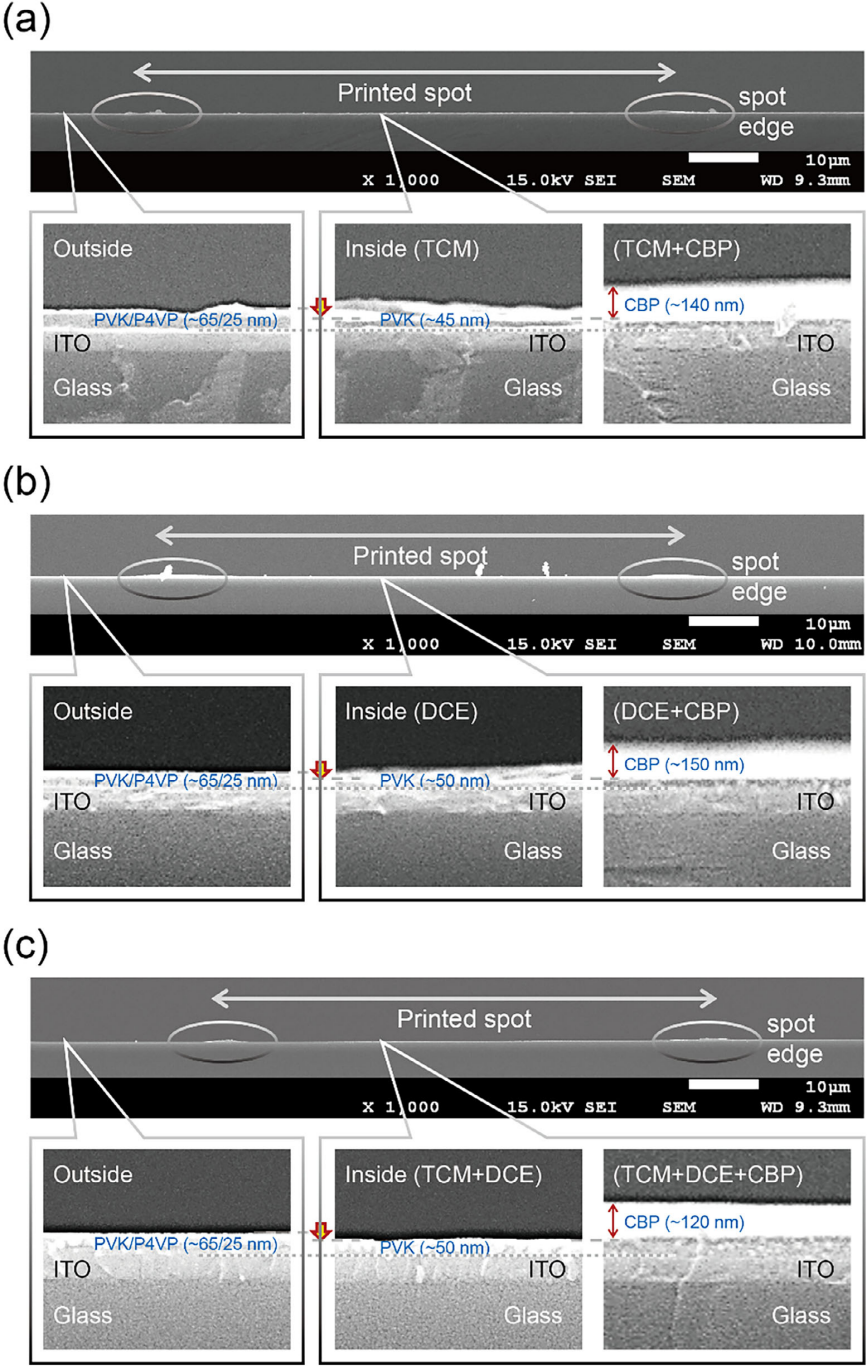
Cross‐sectional SEM characterization of vertical structural selectivity during the micro‐inlay process. Model P4VP (∼25 nm)/PVK (∼65 nm) bilayers on ITO substrates were examined after solvent‐only printing and after the printing of CBP‐containing inks using (a) neat TCM, (b) neat DCE, and (c) mixed TCM:DCE (3:1). In the solvent‐printed regions, the disappearance of the P4VP overlayer together with the retention of a continuous underlying layer (∼45–50 nm) is consistent with preferential removal of the P4VP layer while preserving a continuous underlying transport layer within the spatial resolution of SEM. Continuous CBP‐containing layers subsequently form above the retained underlayer, supporting structural compatibility with the micro‐inlay process.

While Raman mapping confirms the lateral chemical separation between CBP and the displaced P4VP phase, an assessment of the vertical structural evolution of the polymer layers during the micro‐inlay process is also required. To examine this solvent‐dependent vertical restructuring directly, cross‐sectional scanning electron microscopy (SEM) analyses were conducted on model bilayers comprising P4VP (∼25 nm) deposited on PVK (∼65 nm) on ITO substrates (Figure 6). In the unprinted region, the intact bilayer structure is clearly preserved, with the P4VP layer located above the PVK HTL. After the deposition of solvent‐only droplets, the P4VP overlayer is no longer visible in the printed region, while a continuous underlying layer with a thickness of approximately 45–50 nm remains in all three solvent systems, namely neat TCM (Figure 6a), neat DCE (Figure 6b), and TCM:DCE (3:1) (Figure 6c). This observation is consistent with the selective removal of the sacrificial P4VP layer and the concomitant retention of a continuous underlying transport layer assigned to the PVK HTL within the spatial resolution of the present cross‐sectional SEM analysis, although slight thinning or interfacial mixing cannot be fully excluded. When CBP‐containing inks are printed, a continuous CBP‐containing layer forms above this retained underlayer, supporting its structural compatibility with the subsequent layer formation process. These structural observations are consistent with a kinetically controlled interfacial restructuring process rather than the extensive solvent‐induced destruction of the underlying PVK HTL. These results therefore support the solvent‐regulated evaporation pathway proposed in Figure 1, in which controlled swelling and delayed solidification govern selective polymer displacement while maintaining the functional integrity of the transport underlayer.

#### 2.3.3 Nanoscale Homogeneity of the EML Probed by AFM and a HSP Analysis

**FIGURE 7:**
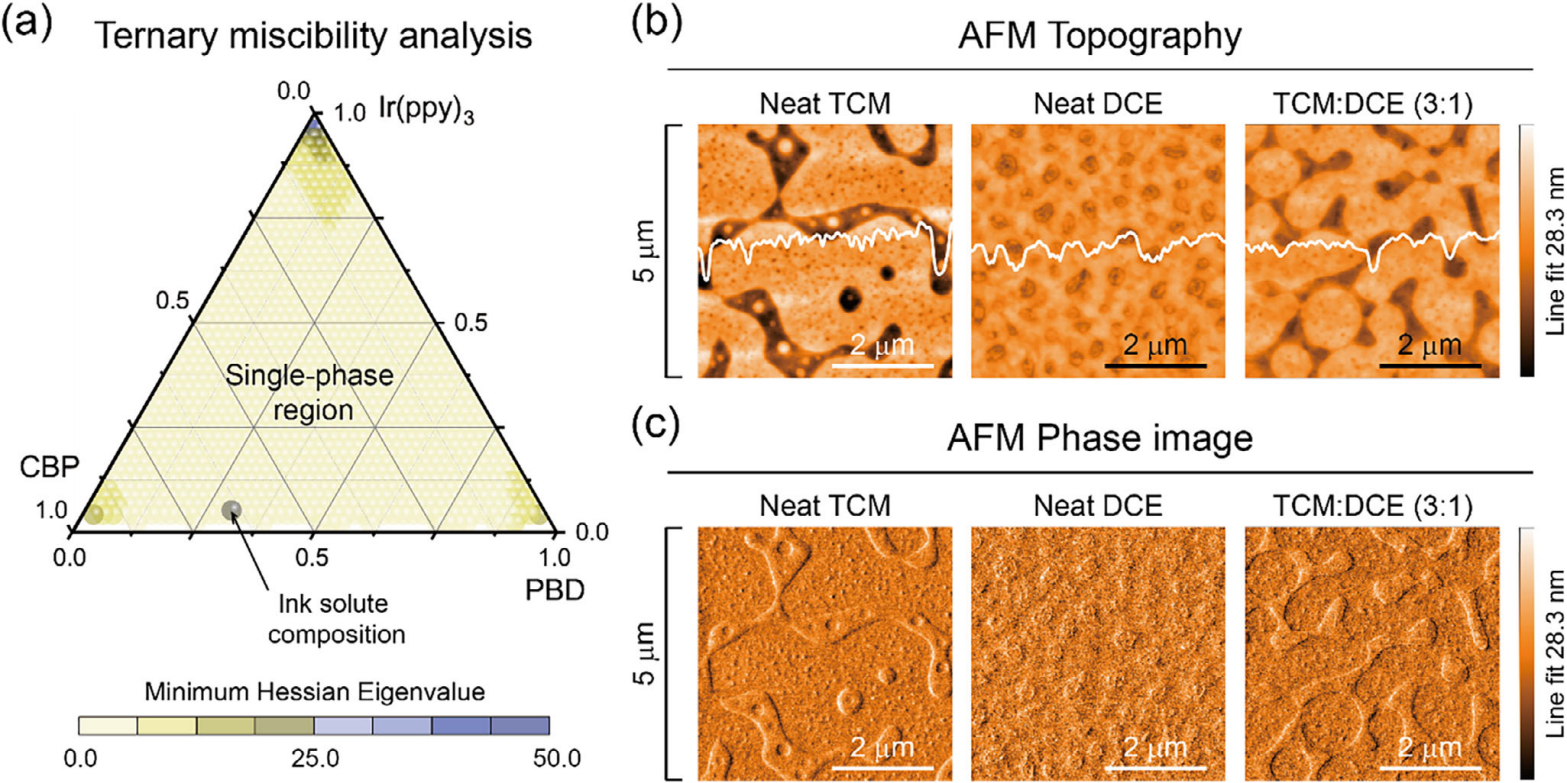
Nanoscale homogeneity of the printed emissive spot: (a) HSP‐based ternary miscibility diagram of the CBP–PBD–Ir(ppy)_3_ system, indicating that the device composition (65:30:5 wt.%) lies within the single‐phase region. (b) AFM topography images (5 × 5 µm^2^, tapping mode) acquired at the center of pixels printed using neat TCM, neat DCE, and TCM:DCE (3:1) solvents (RMS roughness: 5.0, 2.4, and 3.6 nm, respectively). (c) Corresponding AFM phase images from the same regions (RMS phase deviation: 2.3°, 1.5°, and 1.6°). The phase contrast follows the surface topography without revealing distinct nanoscale domains, indicating no detectable solvent‐induced internal phase separation within the measurement resolution.

While Raman mapping and cross‐sectional SEM confirm lateral chemical separation and vertical layer exposure, the nanoscale homogeneity of the emissive pixel interior must also be verified. Because Raman primarily probes the lateral composition, whereas SEM provides microscale structural information along the vertical direction, complementary nanoscale analyses are required to assess whether solvent engineering induces aggregation or compositional heterogeneity within the EML. To examine this point, additional thermodynamic and spatially resolved surface analyses were conducted (Figure 7).

A HSP‐based ternary miscibility assessment of the CBP–PBD–Ir(ppy)_3_ system places the device composition used in this study (CBP:PBD:Ir(ppy)_3_ = 65:30:5 wt.%) within the calculated single‐phase stability region (Figure 7a), indicating no significant thermodynamic driving force for compositional phase separation in the host–dopant mixture [66, 67]. This interpretation is consistent with previous reports showing that intermolecular compatibility critically influences the morphology and device performance of ternary phosphorescent OLED EMLs [66, 67]. Atomic force microscopy (AFM) topography images acquired from the interior region of the printed emissive pixels revealed solvent‐dependent nanoscale surface morphologies (Figure 7b), reflecting differences in the drying kinetics and redistribution behavior during film formation. In contrast to the more pronounced ridge‐like features observed for neat TCM and the finer granular texture obtained for neat DCE, the mixed TCM/DCE solvent produced a comparatively uniform surface morphology within the emissive region. Notably, the RMS roughness values of the CBP–PBD–Ir(ppy)_3_ films in Figure 7 are slightly higher than the *R*_a_ values of pure CBP films in Figure 3, although a direct comparison is limited by differences in the measurement modality, scan area, and roughness definition.

AFM phase images were also acquired from the same scan regions to determine whether these surface features were accompanied by independent nanoscale phase domains (Figure 7c). In tapping‐mode AFM, the phase contrast is generally interpreted in terms of local variations in the tip–sample interactions and nanoscale heterogeneity rather than as a direct compositional map [68, 69]. Across the solvent systems examined here, the phase contrast largely followed the underlying topographic features and did not reveal distinct additional domains decoupled from the surface morphology [68, 69]. Therefore, the combined absence of independent nanoscale phase domains in the AFM images and the thermodynamic single‐phase tendency predicted by the HSP analysis support the conclusion that no detectable solvent‐induced internal phase separation or aggregation occurs within the emissive spot at the spatial resolution of the present measurements. These results indicate that the mixed solvent does not introduce detectable nanoscale phase separation within the emissive spot.

### 2.4 Device Performance and Printing Reliability of Single‐Solvent Inks

**FIGURE 8:**
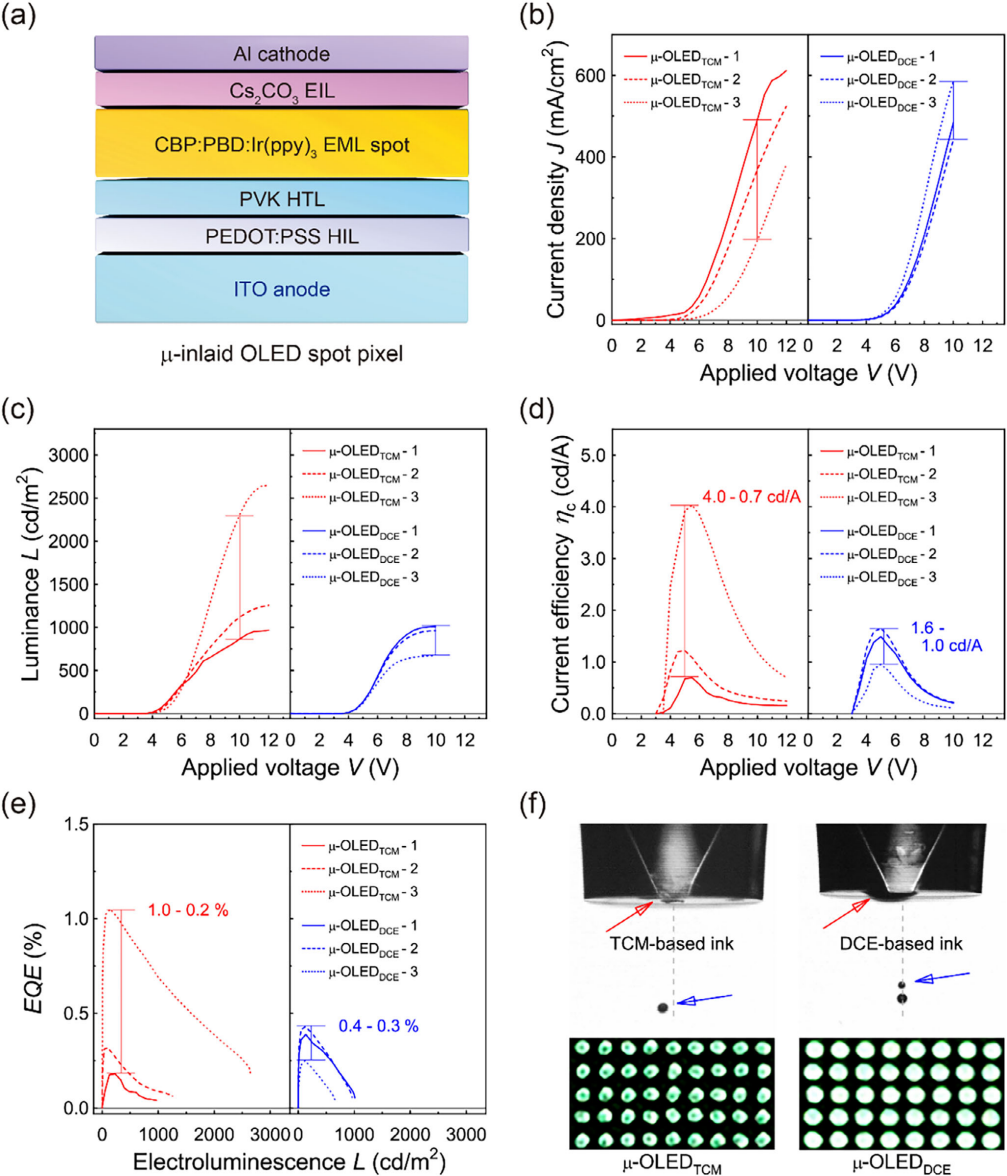
Electrical performance and printability of single‐solvent, inkjet‐defined µ‐OLEDs: (a) Schematic illustration of a µ‐OLED pixel with an EML (CBP:PBD:Ir(ppy)_3_) inlaid into an insulating P4VP layer via the single‐step inkjet printing process. (b–e) Device characteristics of three µ‐OLED arrays fabricated using single‐solvent inks: neat‐TCM‐based ink (left, red) and neat‐DCE‐based ink (right, blue): (b) *J–V*, (c) *L–V*, (d) *η–V*, and (e) *EQE–L*. (f) Top: High‐speed images capturing droplet ejection failures from a 50‐µm‐diameter piezoelectric nozzle for neat‐TCM‐based (left) and neat‐DCE‐based (right) inks. Bottom: Corresponding optical micrographs of 180 dpi green µ‐OLED arrays shown in operation at 10 V (3 × 2 mm^2^).

To evaluate the effects of the solvent carrier on the performance and reliability of µ‐OLED devices, we applied the single‐step micro‐inlay printing strategy to full device stacks. We prepared three otherwise identical emissive inks by dissolving a ternary small‐molecule blend of CBP (host), PBD (electron transporter), and Ir(ppy)_3_ (green emitter) in either neat TCM, neat DCE, or the 3:1 (w/w) TCM/DCE blend. Sub‐nanoliter droplets (approximately 0.11 nL) were deposited at 180 dpi onto substrates consisting of a 25‐nm‐thick P4VP layer on a 65‐nm‐thick PVK HTL and a 40‐nm‐thick PEDOT:PSS HIL on ITO‐coated glass. Solvent‐induced flows, specifically a capillary‐driven flow and a Marangoni back‐flow, enabled the emissive solutes to self‐confine within a 100‐µm‐diameter area. Finally, a 2‐nm‐thick cesium carbonate (Cs_2_CO_3_) electron‐injection layer (EIL) and a 100‐nm‐thick aluminum (Al) cathode were thermally evaporated to complete the bottom‐emitting µ‐OLED structure (Figure 8a). To enable an accurate comparison and minimize variability due to the fabrication conditions, all µ‐OLEDs were co‐fabricated in a single thermal‐evaporation batch. The batch included three types of µ‐OLED arrays, prepared using neat TCM, neat DCE, and the 3:1 (w/w) TCM/DCE blend (see Section 2.5). This controlled process isolates solvent formulation as the only variable, meaning that any observed differences in device behavior arise from the ink chemistry as opposed to variations between batches.

µ‐OLED arrays fabricated with the neat‐TCM‐based ink exhibited clear rectifying behavior in the corresponding current density–voltage (*J–V*) and luminance–voltage (*L–V*) characteristics (see Figure 8b,c), consistent with balanced charge injection and transport through the micro‐inlaid EML. The average device performance, as shown in Figure 8b–e, was characterized by a mean peak luminance (${\bar{L}}_{\max}$) value of (1.6 ± 0.9) × 10^3^ cd m^−2^ at 12 V, mean peak current efficiency (${\bar{\eta }}_{\max}$) of 1.9 ± 1.7 cd A^−1^, and a mean peak *EQE* (${\bar{EQE}}_{\max}$) value of 0.5 ± 0.5%. These values confirm that solvent‐driven self‐confinement is compatible with effective device operation. However, the µ‐OLED arrays exhibited substantial variability, with performance metrics showing relative standard deviations (σs) exceeding 50%. Optical micrographs revealed various defects, including irregular‐shaped pixels, distorted geometries, and luminance non‐uniformity, which contributed to high turn‐on voltages. These defects are attributed to the unstable jetting of the neat‐TCM ink driven by its rapid evaporation and high vapor pressure, in turn perturbing droplet formation and/or the subsequent drying kinetics. In contrast, µ‐OLED arrays printed with the neat‐DCE‐based ink exhibited lower absolute performance but markedly improved array‐to‐array uniformity. The average metrics were as follows: ${\bar{L}}_{\max}$ of (0.9 ± 0.2) × 10^3^ cd m^−2^, ${\bar{\eta }}_{\max}$ of 1.4 ± 0.4 cd A^−1^, and ${\bar{EQE}}_{\max}$ of 0.4 ± 0.1% (see Figure 8b–e). These devices showed poor performance but exhibited a narrow turn‐on voltage window and uniform luminance across the panel. Such improved uniformity is consistent with the favorable rheology of DCE, mainly due to its higher boiling point and lower vapor pressure.

To clarify the mechanism behind these contrasting behaviors, high‐speed imaging of the droplet ejection process was conducted. The neat‐TCM‐based ink frequently exhibited erratic ligament formation, asymmetric breakup, and nozzle clogging due to rapid solvent volatilization (Figure 8f, top left). A transient irregular skin formed at the nozzle tip, intermittently obstructing droplet emission and producing malformed or missing droplets. These ejection anomalies resulted in incomplete or malformed pixels, consistent with the broad scatter in the optoelectronic performance data and the distorted geometry (Figure 8f, bottom left). In contrast, the neat‐DCE‐based ink maintained a stable ejection profile without skin formation, enabling reproducible droplet generation. However, its higher viscosity and lower solubility for the emissive solute blend increased the likelihood of satellite droplet formation and ligament thinning (Figure 8f, top right). These effects occasionally led to underfilled cavities or diluted emissive concentrations, thereby reducing the luminance and efficiency despite the consistent geometry (Figure 8f, bottom right) [70].

These observations highlight a central limitation of single‐solvent formulations: optimizing the jetting stability tends to compromise the quality of the EML. Volatile solvents such as TCM drive strong Marangoni back‐flows and yield high *L*_max_ values, but they are also associated with poor device uniformity due to jetting artifacts. Less volatile solvents such as DCE eject droplets reliably and preserve the geometric consistency, yet at the cost of lower radiative performance. This trade‐off motivated the binary‐solvent strategy investigated here. By combining the strong confinement dynamics of TCM with the stable jetting characteristics of DCE, the mixed‐solvent formulation offers a path toward high pixel fidelity together with robust optoelectronic performance, as discussed in the next section.

### 2.5 Device Performance and Printability: Mixed‐Solvent Effects

**FIGURE 9:**
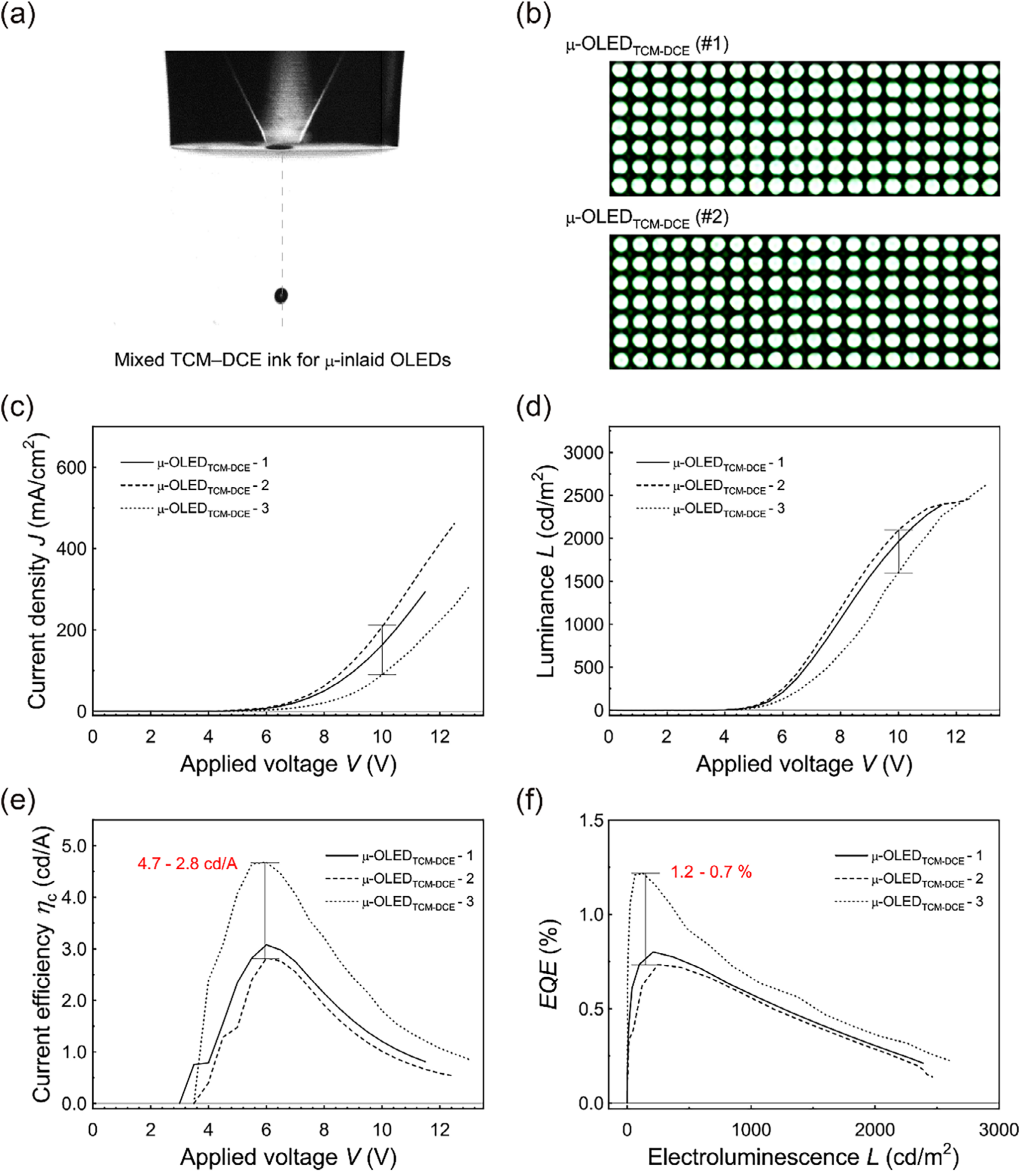
Printability and device performances of µ‐OLED arrays fabricated using a solvent‐programmed binary TCM/DCE ink. (a) High‐speed image of a droplet ejected from a 50‐µm‐diameter piezoelectric nozzle using a TCM/DCE solvent ink mixed at a ratio of 3:1 (w/w). (b) Optical micrographs of two green µ‐OLED arrays operating at 10 V. The arrays have a resolution of 180 dpi and an active area of 3 × 2 mm^2^, with inlaid emissive pixels composed of CBP:PBD:Ir(ppy)_3_ embedded in insulating P4VP layers. (c–f) Device characteristics of three µ‐OLED arrays printed with the TCM/DCE mixed‐solvent ink: (c) *J–V*, (d) *L–V*, (e) *η–V*, and (f) *EQE–L* plots.

To overcome the limitations of single‐solvent formulations, we adopted a binary solvent strategy by blending TCM and DCE at a 3:1 (w/w) ratio. This mixture was designed to combine the strong self‐confinement dynamics driven by TCM with the jetting stability imparted by DCE. High‐speed imaging (Figure 9a) reveals axisymmetric, stable droplet ejection from a 50‐µm piezoelectric nozzle. Unlike neat‐TCM‐ or neat‐DCE‐based inks, the use of which often leads to skin‐induced nozzle blockages or satellite droplet formation, the TCM/DCE blend produces clean ligaments and stable primary droplets with minimal satellites. This stable jetting translates directly to high pattern fidelity, as shown by the optical micrographs in Figure 9b. The resulting µ‐OLED arrays, printed at 180 dpi with 100‐µm pixels, exhibit uniform green emission and consistent jetting across the 3 × 2 mm^2^ active area, with no missing, distorted, or irregularly shaped spots. Importantly, although the geometric outline of individual droplets may exhibit minor edge irregularities depending on the solvent system, electroluminescence predominantly originates from the central emissive region, where charge recombinations occur, rather than from the droplet rim. Therefore, device performance is more directly determined by the thickness uniformity and compositional homogeneity of the active emission region than by geometric edge perfection. In this context, the mixed‐solvent system improves device characteristics not by inducing internal separation of the EML components but by regulating the drying kinetics and preserving a more favorable redistribution pathway during film formation [66, 67]. These results also define the practical scope of the present solvent system. Although introducing the less volatile DCE improves jetting robustness and suppresses the severe drying‐related defects observed with neat TCM, the present study does not claim universal transferability to all commercial printheads or to all higher‐boiling‐point solvent systems, and further validation will be required for such systems. For translation to other printing platforms, solvent selection must satisfy a broader printability window involving not only the evaporation rate, but also the viscosity, surface tension, wetting characteristics, and stable drop formation under the corresponding actuation conditions [58, 59]. In this respect, higher‐boiling‐point solvents may, in principle, offer a longer liquid‐phase window, but excessively slow evaporation may also reduce pattern fidelity and throughput. Therefore, the present results should be interpreted as an experimentally validated proof‐of‐concept for the TCM/DCE system, whereas the generalizable element is the drying‐kinetics‐guided solvent‐design strategy. It was also noted that when the TCM:DCE weight ratio departs from the 3:1 optimum level, the mixture increasingly behaves as a single solvent, leading to non‐uniform ink spots and/or printing defects in the inkjet‐printed arrays.

The mixed‐solvent formulation demonstrates clear performance advantages over either neat solvent alone. Figure 9c–f shows the consistent device characteristics of three nominally identical µ‐OLED arrays fabricated with the 3:1 (w/w) TCM/DCE ink. The devices exhibit a turn‐on voltage near 3.5 V and a ${\bar{L}}_{\max}$ value reaching (2.4 ± 0.1) × 10^3^ cd m^−2^ at 11.5 V. These results are approximately 1.4‐ and 2.7‐fold higher than those from neat‐TCM‐ and neat‐DCE‐based devices, respectively. The devices also show a narrow performance spread. With a high ${\bar{L}}_{\max}$ value of 2.4 × 10^3^ cd m^−2^, the standard deviation σ for the µ‐OLED arrays is just 76 cd m^−2^, representing a significant improvement in uniformity. In comparison to neat‐TCM‐ and neat‐DCE‐based arrays (Figure 8), which have corresponding σ values of approximately 900 and 180 cd m^−2^, the mixed‐solvent formulation substantially reduces the variability among arrays. Moreover, the efficiency metrics of the mixed‐solvent devices show a similar favorable trend. For these devices, ${\bar{\eta }}_{\max}$ becomes 3.5 ± 1.0 cd A^−1^, while ${\bar{EQE}}_{\max}$ is 1.0 ± 0.3%. These values outperform those of neat‐TCM‐ink‐based devices (${\bar{\eta }}_{\max}$ = 1.9 ± 1.7 cd A^−1^, ${\bar{EQE}}_{\max}$ = 0.5 ± 0.5%) and neat‐DCE‐ink‐based devices (${\bar{\eta }}_{\max}$ = 1.4 ± 0.4 cd A^−1^, ${\bar{EQE}}_{\max}$ = 0.4 ± 0.1%).

These superior performance outcomes provide strong evidence for the effectiveness of the solvent‐programmed micro‐inlay strategy. In particular, the interior profile observed under the TCM/DCE condition suggests a more favorable thickness distribution of the CBP‐based EML within the central recombination zone. The improved performance of the mixed‐solvent devices is also consistent with the cross‐sectional SEM observations in Figure 6, which indicate selective removal of the P4VP layer while preserving the underlying transport layer within the resolution of the present analysis. Together with the spatial Raman mapping and fully operational *J–V–L* characteristics, these results support solvent‐induced emissive confinement rather than destructive removal of the underlying functional layers. Collectively, these findings indicate that the TCM/DCE formulation improves device operation primarily by preserving the functional uniformity of the central emissive region, while also enabling uniform, high‐performance µ‐OLED arrays.

### 2.6 Influence of Solvents on the Uniformity and Performance of µ‐OLEDs

To quantify the impact of the solvent composition on the performance and reliability of µ‐OLED devices, we introduce two composite metrics: (i) a pixel‐wise luminance uniformity index (*U*_L_) with the coefficient of variation (*CV* = standard deviation σ / mean luminance $\bar{L}$) for µ‐OLED pixels using *U*_L_ ≡ *1/CV* $=\bar{L}\;/\sigma$, which increases with the pixel‐to‐pixel luminance uniformity and statistical reproducibility; and (ii) a performance figure of merit (*FoM* ≡ *L*_max_ × *EQE*_max_​), which captures the absolute array device performance. The pixel‐to‐pixel luminance uniformity *U*_L_ was evaluated using the open‐source ImageJ platform to derive the mean luminance of each spot pixel, followed by mean luminance normalization at the given applied voltage (here, an applied voltage of 8 V).

**FIGURE 10:**
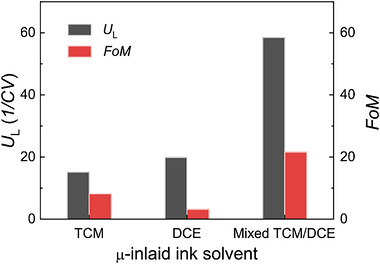
Luminance uniformity and performance *FoM* outcomes for µ‐OLED arrays fabricated with different carrier solvents. The bar plots show the uniformity index *U*_L_ (1/*CV*; larger values denote better pixel‐to‐pixel uniformity) and the device performance *FoM* (*L*_max_ × *EQE*_max_). For each array, *U*_L_ was estimated from the pixel‐wise luminance *CV* evaluated over ≥ 300 pixels per array. Each array comprises inlaid emissive pixels of CBP:PBD:Ir(ppy)_3_ embedded in a P4VP matrix inkjet‐printed using a solvent of either neat TCM, neat DCE, or the 3:1 (w/w) TCM/DCE blend.

Figure 10 presents a comparative analysis of µ‐OLED arrays printed with the three solvent systems. The arrays printed with neat‐TCM inks exhibit the lowest uniformity index of the pixels (*U*_L_ ≈ 15.2), indicating significant pixel‐to‐pixel luminance variability. This inconsistency is attributed to the high volatility of TCM, which leads to jetting instability, erratic jetting, intermittent droplet drop‐outs, and malformed or missing pixels. Despite these drawbacks, the TCM‐based arrays reach a *FoM* of about 8.2, driven by high peak brightness in well‐formed micro‐inlaid pixels. Switching to neat‐DCE inks markedly improves the pixel uniformity index (*U*_L_ ≈ 19.9) due to the higher boiling point of DCE and its lower evaporation rate, which together stabilize the jetting characteristics. However, the *FoM* of the DCE‐based arrays decreases to approximately 3.1 because the lower solute concentration and weaker Marangoni‐flow‐driven confinement as the droplet dries reduce both the luminance and efficiency. These outcomes illustrate the intrinsic trade‐offs in single‐solvent systems, where optimizing the stability often compromises the radiative performance. In contrast, the TCM/DCE blend with a 3:1 (w/w) ratio outperforms both neat solvents in all key parameters: specifically, *U*_L_ increases to approximately 58.5, which is about 3.9‐ and 2.9‐fold higher than the corresponding values for neat‐TCM‐ and neat‐DCE‐based devices, respectively, and the *FoM* reaches approximately 21.6, representing about 2.6‐ and 6.9‐fold increases over the corresponding values of neat‐TCM‐based and neat‐DCE‐based devices, respectively. This improvement stems from the synergistic tuning of the jetting and phase‐separation dynamics; the blend utilizes the rapid Marangoni back‐flow of TCM to achieve dense central emitter accumulation and the rheological stability of DCE to suppress satellite droplets and jetting instability. These combined effects produce tightly confined, compositionally pure micro‐inlaid pixels with minimal variation in the brightness, geometry, and efficiency.

**FIGURE 11:**
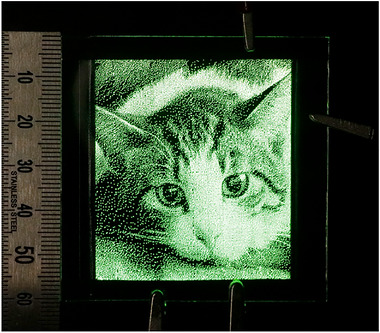
Photograph of an operating large‐area µ‐inlaid OLED array (driven at 10.0 V) on ITO‐coated glass with a P4VP layer (25 nm). The array was fabricated by single‐step inkjet micro‐inlay printing at 180 dpi using the TCM/DCE (3:1 w/w) blended solvent, yielding uniform green electroluminescence from self‐organized micro‐inlaid EML spots across the 6.0 × 6.0 cm^2^ substrate.

To assess the scalability of the proposed method, a 6.0 × 6.0 cm^2^ ITO‐coated glass substrate bearing a P4VP layer (25 nm) was patterned by the solvent‐programmed, single‐step inkjet micro‐inlay process (Figure 11). The printed array comprises densely packed, self‐organized micro‐inlaid EML spots that emit uniform green electroluminescence under bias. The photograph demonstrates that complex, high‐resolution pixel patterns can be generated without photolithography. The uniform brightness and low pixel‐to‐pixel variation across the field indicate high pattern fidelity and good process uniformity, with no pinholes or misalignment. These results support the scalability and run‐to‐run reproducibility of the inkjet‐inlaid architecture and underscore the role of phase‐separable material systems and favorable interactions among the TCM/DCE blend, the small‐molecule emitters, and the P4VP underlayer in enabling precise micro‐inlay formation. Overall, this approach provides a cost‐effective, scalable pathway toward high‐resolution, high‐performance µ‐OLED displays.

Collectively, these results validate the central premise of the solvent‐programmed inkjet micro‐inlay process: binary‐solvent engineering with a highly volatile TCM/lower‐volatility DCE blend yields significant improvements in the luminance, current efficiency, *EQE*, and pixel‐to‐pixel uniformity of µ‐OLEDs. These findings further demonstrate that judicious solvent blending enables concurrent control of droplet‐scale fluid mechanics and polymer‐solute‐solvent thermodynamics. While the present study establishes a practical benchmark for inkjet‐printed µ‐OLEDs, further performance gains are attainable. Tighter control of the interfacial swelling, droplet‐impact kinetics, and polymer dissolution can be achieved through architectural modifications. These include the selection of the host–guest emitter system, the optimization of HSP windows for carrier‐solvent blends, and the introduction of charge‐transport layer stacks. Preliminary trials indicate that these strategies can significantly improve performance capabilities, with luminance levels exceeding 4000 cd m^−2^, current efficiencies surpassing 20 cd A^−1^, and *EQE*_max_ levels exceeding 4% (data not shown here). However, such modifications were intentionally excluded from this study to isolate the intrinsic effect of the solvent formulation on device performance metrics. By integrating patterning and material deposition via solvent‐driven phase separation into a single‐step printing process, this study establishes solvent‐programmed inkjet micro‐inlay printing as a practical and effective engineering route for scalable, high‐resolution OLED displays and other solution‐processed optoelectronic devices [71, 72, 73].

## 3 Conclusion

In summary, this study identifies mixed‐solvent engineering as a pivotal route to the scalable, high‐fidelity inkjet patterning of µ‐inlaid OLED pixels. Transitioning from single‐ to binary‐solvent formulations provides synergistic control over droplet dynamics, the interfacial morphology, and pixel‐level uniformity. These three interdependent parameters critically influence device performance, fabrication yields, and the manufacturability of high‐resolution µ‐OLED arrays. A blend of TCM and DCE at a 3:1 (w/w) ratio leverages complementary physicochemical traits: the rapid evaporation and strong Marangoni back‐flow from TCM together with the higher viscosity and lower volatility of DCE. This optimized blend produces satellite‐free, axisymmetric droplets with controlled spreading and localized interaction with a phase‐separable P4VP layer, yielding a consistently confined emissive domain with a diameter of approximately 100 µm and with surface roughness below 2 nm in a single inkjet step. Spatially resolved micro‐Raman mapping, cross‐sectional SEM, and AFM analyses collectively indicate that the binary‐solvent system promotes lateral phase separation between the CBP emissive region and the displaced P4VP phase, while preserving the underlying transport layer and maintaining nanoscale homogeneity within the emissive spot. Coupled with high droplet fidelity, these results confirm that the binary carrier enables uniform, defect‐free deposition across the substrate. These interfacial and fluidic optimizations markedly improved device performance: specifically, µ‐OLEDs printed using the 3:1 (w/w) TCM/DCE blend exhibited an *L*_max_ of (2.4 ± 0.1) × 10^3^ cd m^−2^, a *η*_max_ of 3.5 ± 1.0 cd A^−1^, and an *EQE*_max_ of 1.0 ± 0.3%. Using the unrounded *L*_max_ and *EQE*_max_ values, the performance *FoM* (*L*_max_ × *EQE*_max_) reached approximately 21.6, corresponding to about 2.6‐ and 6.9‐fold increases over the corresponding values for the neat‐TCM‐ and neat‐DCE‐based devices, respectively. Furthermore, the pixel‐wise *U*_L_ index increased to approximately 58.5, demonstrating improvements that exceed by approximately 3.9‐ and 2.9‐fold those of devices printed with neat‐TCM‐ and neat‐DCE‐based inks, respectively. Overall, mixed‐solvent engineering offers a rational route to optimize compatibility among the solvent, polymer, and solute, thereby controlling lateral phase separation, the deposition morphology, pixel uniformity, and the optoelectronic performance. The resulting solvent‐programmed inkjet micro‐inlay process is lithography‐free, scalable, and intrinsically compatible with sub‐100‐µm pixels, multi‐color (RGB) emitters, and flexible or three‐dimensional substrates. These advantages establish this approach as a versatile and scalable platform for next‐generation high‐resolution OLED displays and other solution‐processed optoelectronic applications.

## 4 Experimental Section/Methods

### 4.1 Materials

P4VP (M_w_ ≈ 60 000), N,N′‐bis(3‐methylphenyl)‐N,N′‐diphenylbenzidine (TPD), PVK, PBD, and Cs_2_CO_3_ were purchased from Sigma–Aldrich. Ir(ppy)_3_ and CBP were obtained from Luminescence Technology Corp. (Lumtec). The aqueous dispersion of PEDOT:PSS (Clevios P VP AI 4083) was supplied by H.C. Starck. All reagents were used as received without further purification.

### 4.2 Film and Device Fabrication

Pre‐patterned ITO coated on glass substrates with a thickness of 80 nm and sheet resistance of 20 Ω/sq served as the transparent anodes for µ‐OLED fabrication. The ITO substrates were cleaned sequentially by ultrasonication in ethanol, a detergent solution, and deionized water, after which they were dried with nitrogen gas. Immediately before use, the substrates were treated with ultraviolet ozone for 5 min to remove residual organic contaminants and enhance the surface wettability. Next, a 40‐nm‐thick HIL of PEDOT:PSS was spin‐coated onto the ITO and thermally annealed at 120°C for 20 min. Then, a 65‐nm‐thick HTL was deposited by spin‐coating a chloroform‐based solution containing TPD and PVK at a weight ratio of 6:7, with this followed by baking at 50°C for 2 min. A 25‐nm‐thick P4VP insulating layer was formed by spin‐casting a 0.5 wt.% P4VP solution in isopropyl alcohol onto the HTL. All subsequent steps were performed in a nitrogen‐filled glovebox. Inkjet printing was carried out using an Omnijet 100 system equipped with a 50‐µm piezoelectric nozzle (MicroFab). Two types of ink were prepared for inkjet printing: (i) a control ink containing only CBP, and (ii) a green‐emissive ink containing CBP, PBD, and Ir(ppy)_3_ dissolved in either neat TCM, neat DCE, or a TCM/DCE mixture at a 3:1 (w/w) ratio. Sub‐nanoliter droplets were dispensed directly onto the P4VP‐coated substrates to generate localized EMLs via self‐confinement and lateral phase separation. After inkjet printing, the µ‐OLED devices were fabricated by thermally evaporating a 2‐nm‐thick Cs_2_CO_3_ EIL and a 100‐nm‐thick Al cathode. All vacuum deposition processes were conducted under a base pressure below 2.0 × 10^−6^ Pa. The final µ‐OLEDs had an active emission area of 3 × 2 mm^2^.

### 4.3 Film and Device Characterization

Three‐dimensional height profiles and surface roughness of the spin‐coated films and printed pixels were evaluated using a non‐contact optical surface profiler (NV‐2400, Nanosystem Co., Ltd.). The chemical composition and spatial phase separation in the printed regions were examined using a confocal Raman microscope (Alpha300, WITec) with a spectral resolution of 1 cm^−^^1^ and a lateral resolution of 200 nm. The microscopic morphology and cross‐sectional structure of the fabricated layers were further examined by high‐resolution SEM (JSM‐6700F, JEOL Ltd.). Samples were cleaved perpendicular to the substrate surface to expose the multilayer cross‐section, and the thicknesses of the P4VP, PVK, and printed CBP‐containing layers were determined from calibrated SEM micrographs. The surface morphology of the spin‐coated films and printed pixels was also characterized by AFM (FlexAFM, Nanosurf AG). AFM measurements were performed in tapping mode to acquire topography and phase images from identical scan regions, enabling direct comparison between surface morphology and local phase contrast. Silicon cantilevers with a nominal resonance frequency of approximately 300 kHz were used for all measurements. AFM images were typically acquired over a scan area of 5 × 5 µm^2^ at the center of the printed emissive pixels to minimize rim‐induced morphological artifacts near the pixel boundary. The current density–luminance–voltage (*J–L–V*) characteristics of the µ‐OLED arrays were measured using a source meter (Keithley 2400) and a chromameter (CS‐2000, Konica Minolta). All electrical and optical measurements were conducted under ambient laboratory conditions at room temperature.

The pixel‐to‐pixel luminance uniformity of the µ‐OLED pixels was analyzed using ImageJ software (ver. 1.54). For spatial calibration, the mean center‐to‐center spacing measured in the image was assigned a value of 141 µm (180 dpi) in the software. Each field comprised a 16 × 20 array of circular pixels (dot diameter ≈ 100 µm; field size ≈ 3 × 2 mm^2^; N = 320). Pixels were segmented in the software to obtain centroids, and intensities were measured with fixed circular apertures with a radius of 50 µm for all dots (half the dot diameter; optional narrow background annulus for median subtraction). The per‐dot luminance *L*_i_ was obtained by mean‐normalization, with $L_{i}=g_{i}\;\cdot \;\bar{L}\;/\;\bar{g}$, where *g*_i_ is the raw per‐dot mean, $\bar{g}$ is the across‐pixel mean, and $\bar{L}$ is the calibrated field mean. Reported metrics consist of the standard deviation σ, the coefficient of variation *CV* = σ/$\bar{L}$, and the uniformity level *U*_L_ ≡ *1/CV*, with 95% confidence intervals estimated by a nonparametric bootstrap method.

### 4.4 Simulation of Evaporation Kinetics of Neat and Binary Solvents

For the evaporation analysis shown in Figure 1b, a simplified model based on Raoult's law was used to estimate the time evolution of the solvent composition and the normalized evaporation behavior of neat TCM and the 3:1 (w/w) TCM/DCE binary solvent under a pinned contact‐line condition [49, 52]. The model assumes diffusion‐limited evaporation of a sessile droplet and treats the mixed solvent as a binary liquid in which the more volatile TCM evaporates preferentially, thereby changing the instantaneous liquid composition during the drying process. For a numerical evaluation, the initial droplet volume was set to *V*_0_ ≈ 0.10 nL and the initial contact radius to *R*_0_ ≈ 50 µm, consistent with the printed pixel dimensions (∼100 µm in diameter). The binary solvent was treated as an ideal solution obeying Raoult's law, enabling the calculation of time‐dependent mole fractions and the visualization of the compositional pathway in the ternary diagram (Figure 1c). The resulting compositional evolution was then mapped onto a conceptual ternary pathway in the TCM–DCE–CBP phase space to identify the relative point at which the drying trajectory approaches the solidification boundary.

To evaluate the effective liquid‐processing window more realistically, a threshold‐extension analysis was also performed. Rather than defining the drying endpoint solely by complete solvent depletion, the effective solidification time was operationally defined as the point at which the residual total‐solvent fraction reached a prescribed threshold value, because practical morphology fixation is expected to occur before complete drying in the strict mathematical sense. Comparable threshold‐based approaches have been employed in evaporation‐driven morphology modeling studies. Threshold values of 20%, 15%, 10%, and 5% of the remaining solvent fraction were applied to define the effective liquid‐state window. Based on this analysis, the effective liquid‐state lifetimes for neat TCM were estimated to be 8.01, 8.51, 9.12, and 9.50 s, respectively, whereas those for the TCM/DCE mixture were 10.86, 11.66, 12.83, and 13.83 s. Accordingly, the binary solvent extends the effective liquid‐state lifetime by 2.85–4.33 s, with a representative extension of 3.71 s at the 10% threshold. These values were used to interpret the delayed solidification pathway and the increased opportunity for internal fluid redistribution in the mixed‐solvent system.

## Conflicts of Interest

The authors declare no conflicts of interest.

## Data Availability

The data that support the findings of this study are available from the corresponding author upon reasonable request.
